# Investigating the role of health information technology in the control and management of Systemic Lupus Erythematosus (SLE): a systematic review

**DOI:** 10.1186/s12911-022-02009-y

**Published:** 2022-10-08

**Authors:** Khadijeh Moulaei, Elham Rajaei, Leila Ahmadian, Reza Khajouei

**Affiliations:** 1 Department of Health Information Sciences, Faculty of Management and Medical Information Sciences, Kerman University of Medical Sciences, Kerman, Iran; 2 Department of Internal Medicine, Ahvaz Jundishapur University of Medical Sciences, Ahvaz, Iran

**Keywords:** Systemic Lupus Erythematosus, SLE, Lupus, Health information technology, HIT

## Abstract

**Background:**

Despite the use of health information technology (HIT) for controlling and managing lupus, its effectiveness has not been well studied. The objective of this study was to investigate the role of HIT in controlling and managing lupus.

**Methods:**

We searched Scopus, PubMed, Web of Science, and Embase, using "self-management", "self-care" and "Systemic Lupus Erythematosus" keywords. Two researchers selected relevant papers and extracted data using a data collection form. Disagreements were resolved in consultation with the third and fourth researchers. After extraction, the data were analyzed.

**Results:**

Totally, 23 papers met the inclusion criteria. About 75% of the studies used web and telephone-based technologies. Most services provided with health technologies were ‘Training’ and ‘consulting’. The ‘lifestyle" and ‘Consultation and education’ axes were the most widely used HIT services to control and manage lupus. While, ‘Better management and control of the disease’, ‘Increasing knowledge and awareness of people about lupus’ and ‘Improving behaviors and attitudes toward self-management and self-care’ were also the most important outcomes. ‘Collectiing patient data and information’, 'Providing education and consultation services to patients', 'Measuring patient-reported outcomes', and 'Increasing patients' knowledge and awareness of their disease' were the most important advantages of various technologies. 'Slow internet speed' and 'Challenges and problems related to appearance and usability' and 'Patient concerns about privacy and misuse of their data' were three disadvantages of technologies.

**Conclusion:**

The findings showed that HIT can improve the management and control of lupus and facilitate self-efficacy, self-care, and self-management in patients. The axes and data elements identified in this study can be the basis for developing and implementing efficient HIT-based systems to improve, control, and manage lupus.

## Background

Systemic Lupus Erythematosus (SLE) is an autoimmune, multi-system, chronic, inflammatory, and fatal disease, with different clinical manifestations [1], that has been identified as a common and significant health challenge worldwide [2]. This disease is a disorder of unknown origin that affects several organs and causes various tissue harms by producing and depositing autoantibodies and pathogen immune complexes in tissues and cells [3, 4].

Systemic Lupus Erythematosus has adverse effects on various physical, mental, and social dimensions of patients' health and reduces their quality of life [5, 6]. People with this disease have low self-confidence in dealing with various issues, always feel worried about death, and the nature of their disease is not understood by family and friends [7]. To cope with these issues, like in any other chronic disease, these patients should be able to control the manifestations and physical and mental complications of their disease [8]. One of the methods to control, manage, and combat chronic diseases is the use of health information technology (HIT) [9]. Tani et al.[10] showed the application of HIT for the diagnoses, treatment, prognosis, and management of diseases in various fields of medicine including rheumatic diseases such as lupus erythematosus. The use of HIT has been introduced as a fundamental effort to improve the provision of health services, reduce health care costs and improve the quality of health care [11]. Evidence has shown that health information technologies can improve efficiency and safety in providing health services [12] and introduce opportunities for disease diagnosis, management, and treatment [13, 14]. Also, they can revolutionize the delivery of health care services, reduce medical errors, increase people's understanding of their illnesses, and save their lives [15]. Advances in HIT have provided approaches that support effective and worthwhile health care services and training. For example, mobile technologies, computers, e-mail, and other Internet-based tools have played an important role in improving the management of chronic diseases by supporting clinical decision-making and facilitating patient self-management [16]. In addition, with the use of technologies, patients can communicate with medical professionals about any health conditions, avoiding hospital visit [17].

To our knowledge, so far no systematic study has identified the impact of health information technologies on the control and management of Systemic Lupus Erythematosus. Only two systematic reviews studied health information technologies for the management of Systemic Lupus Erythematosus [10, 18]. These studies have focused only on the role of m-health in the management of Systemic Lupus Erythematosus and have not examined the impact of other technologies. The aim of this study was to investigate the role of health information technology in the control and management of Systemic Lupus Erythematosus.

## Methods

The present study is a systematic review study that has been conducted and reported based on PRISMA checklist [19].

### Search strategy

Four Scopus, PubMed, Web of Science, and Embase databases were searched without time limitation until July 2, 2022 to find the relevant articles. These databases were searched using “Self-management”, “Self-care” and “Systemic Lupus Erythematosus” keywords. The “Self-management” and “Self-care” keywords were combined with the OR operator. Then, they were combined with “Systemic Lupus Erythematosus” by the AND operator. This search strategy was developed by two researchers (KHM and RKH) and finally approved by LA and ER. To search the Scopus database, these keywords were placed in double quotation.

Also, to prevent missing the relevant studies, the reference lists of relevant systematic review studies were examined [10, 18].

### Eligibility criteria

In this study, articles were included that addressed the effect of information technology on self-care and self-management of Systemic Lupus Erythematosus, published in English, and focused on human. Studies that did not focus on the role of health technology in the management and control of SLE were excluded. Also, books, book chapters, letter to the editors, and the abstract of conference articles were excluded.

### Study selection

First, the abstracts of all related articles were retrieved from four scientific databases and entered into Endnote. Duplicate articles were excluded from the study. Then, one of the authors (KHM) reviewed the title, abstract, and keywords and selected the relevant articles according to the inclusion and exclusion criteria. All validated articles were reviewed and finalized by RKH and LA. After final approval of the articles, to extract information the full texts of the articles were reviewed by two researchers, KHM and RKH. Data collection was done using a data extraction form. The validity of this form was confirmed by two medical informatics specialists and a software engineer. Data extraction form included fields such as reference, country, study year he, setting (inpatient/outpatients and academic/non-academic), study type, objectives, sample size, study group (male/female), the mean age of the subjects, time, the device or technology used, the services provided with each technology, the information-educational needs addressed by the technology, duration of the follow-up and the outcomes of using each technology. Finally, RKH and ER re-examined and validated all findings obtained in the data extraction form.

Wherever the required information such as the type of study was not mentioned, we contacted the authors of that study and asked them to provide us with the relevant information.

Moreover, we used descriptive statistics (percentage and frequency) to analyze the data in SPSS 23.0.

## Results

**Fig. 1:**
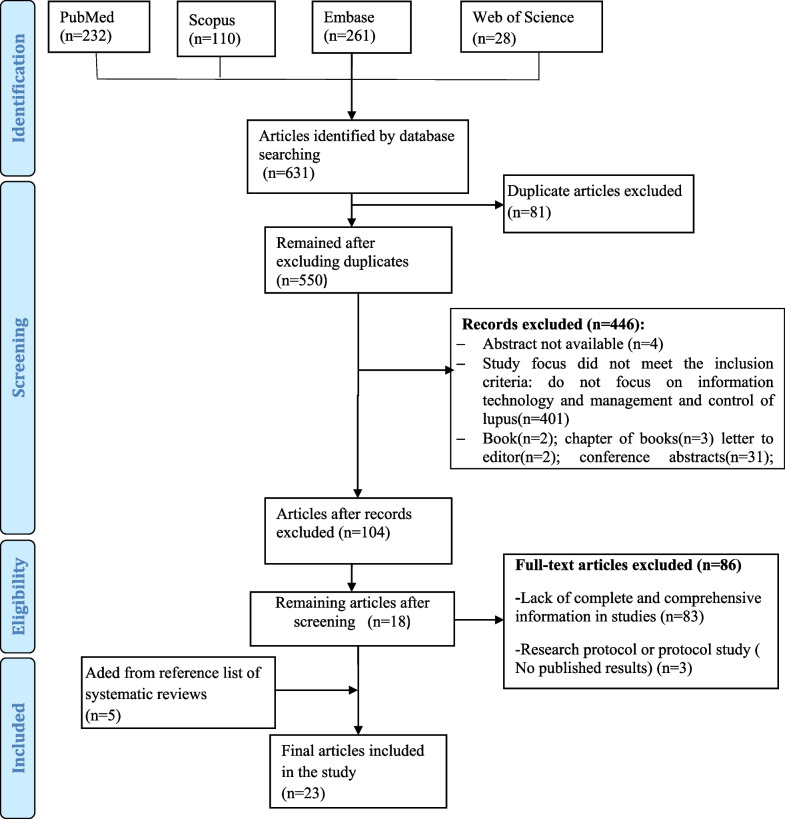
Flowchart classification and resource selection

A total of 631 studies were retrieved from the four databases. Eighty-one duplicate articles were excluded from the study. Then, the remaining 550 articles were carefully examined based on inclusion and exclusion criteria. Finally, 23 articles were included in the study (20 articles from four databases and 5 articles from the reference list of two systematic reviews [10, 18]). The results of this process are shown in Fig. 1.

Table 1 shows the results of reviewing these studies. Most of the studies that used health information technology to manage and control lupus were conducted in the United States (n = 18) [20–37]. The oldest study using information technology to monitor lupus was also conducted in this country [38]. Cohort studies [20, 22, 31, 32] (n = 4), pilot studies [28, 30, 38, 39] (n = 4), and randomized controlled trials (RCT) (n = 4) [36, 40] were the most frequent types of studies in this review. Table 1 shows the information extracted from the studies.

**Table 1:** The main findings of the literature review

Refs.	Country	Study year	Study objective	Setting		Type of study	Sample size	Study group		Age group or mean age	Duration of follow-up	Type of information technology
				Inpatient/outpatients	Academic/non-academic			Man	Woman			
[38]	UK	2002	Development and evaluation of a Web-based educational program for lupus patient information	Outpatients	Academic/non-academic	Pilot study	20–30	√	√	21–50	24 months	Web-based educational program
[20]	USA	2009	Comparing differences in long-term results between adults with childhood-onset lupus and with adult-onset SLE	Outpatients	Non-academic	Cohort study	885	√	√	18 years and younger	1-year	Telephone
[21]	USA	2014	Development of a mobile-based app for adolescents with SLE	Outpatients	Non-academic	Not mentioned	18			16–59	2 days	Mobile-based APP
[22]	USA	2014	Investigation and analysis of the burden of lupus on employment and work productivity	Outpatients	Academic/non-academic	Cohort study	689	√	√	18–64	Not mentioned	Web-based registry
[23]	USA	2016	Acquiring objective measuring of physical activity (PA) using an accelerometer and estimations of energy expenditure based on the self-reported International Physical Activity Questionnaire (IPAQ), and to describe their relationship	Outpatients	Academic/non-academic	Cross-sectional study	129	√	√	18–65	Over 7 days	Wearable accelerometers
[24]	USA	2016	Evaluation of the quality of life of patients with SLE using questionnaires from the Patient-Reported Outcomes Measurement Information System (PROMIS) and quality of Life in Neurological Disorders (Neuro-QoL)	Outpatients	Academic	Not mentioned	333	√	√	≥ 18 years old	3 months	Web-based program for self-reported status (PROMIS)
[25]	USA	2016	Development and usability evaluation of the web-based e-Health tool to facilitate Lupus control management	Outpatients	Academic	Not mentioned	43		√	43.6	2 weeks	Web-Based e-Health Tool
[26]	USA	2017	Investigating the feasibility and potential benefits of peer mentoring to modify disease self-management and quality of life in a patient with SLE	Outpatients	Academic	Feasibility study	450		√	18 years of age or older	12 weeks	Telephone
[39]	UK	2017	Surveying self-reports of SLE patients to determine specific subpopulations susceptible to disease state	Outpatients	Non-academic	Pilot study	80		√	18 or older	2-week	Web-based survey and social media
[27]	USA	2018	Providing knowledge and insight into the experiences of African-American women with SLE through a telephone-based peer mentoring intervention	Outpatients	Academic	Qualitative study	27		√	35–44	12-week	Telephone
[28]	USA	2018	Examining medication adherence in adolescents and young adults with lupus	Outpatients	Non-academic	Pilot study	37		√	13–23	8 week	Web -based educational program
[29]	USA	2018	Investigating the feasibility of PROMIS computerized adaptive tests in lupus outpatients	Outpatients	Academic	Feasibility study	238	√	√	40.6 years	Over 13 months	Web-based program for self-reported status (PROMIS)
[30]	USA	2019	Assessing the cost of the Peer Approaches to Lupus Self-management (PALS) intervention and specifying its effectiveness when compared to existing treatments	Outpatients	Academic	Pilot study	27		√	18 years of age or older	12-week	Telephone
[31]	USA	2019	Comparing major depression hazards among young adults with lupus, and specifying demographic and health-related predictors of depression by self-reported depressive symptoms	Outpatients	Non-academic	Cohort study	546	√	√	18–45	12 Years	Telephone
[32]	USA	2019	Psychometric assessment of the National Institutes of Health(NIH) PROMIS in a multi-racial and multi-ethnic lupus	Outpatients	Academic/non-academic	Cohort study	431	√	√	46.6	Not mentioned	Web-based program for self-reported health status (PROMIS)
[33]	USA	2019	Implementation and distribution of an African American popular opinion web-based e-learning model to improve lupus awareness	Outpatients	Academic/non-academic	Not mentioned	37	√	√	57	four weekly 2–3-h sessions over 1 month	Web-based e-learning
[34]	USA	2020	Reviewing and analyzing usefulness of cellular text messaging for improving adherence among patients with lupus	Outpatients	Academic	Intervention study	70	√	√	13–25	14 months	Mobile-based text reminders
[40]	Canada	2020	Investigating the effectiveness of a physical activity counseling program using a wearable tracker in people with lupus	Outpatients	Non-academic	Randomized controlled trial (RCT)	110	√	√	53.5	24 months	Wearable tracker
[41]	Thailand	2020	Assessing mental health state and exploring causes associated with the disease-specific quality of life among Lupus patients	Outpatients	Non-academic	Cross-sectional study	344	√	√	26.3	4 weeks	Web-based program
[35]	USA	2021	Development and usability evaluation of an e-toolkit designed to supply skills and knowledge about self-management behaviors for individuals with systemic lupus erythematosus	Outpatients	Academic	Not mentioned	15	√	√	Under 25 years old and over 45 years old	Not mentioned	Web-based e- e-dashboard
[36]	USA	2021	Evaluating the effect of high-intensity periodic education with the help of smartphones in patients with SLE	Outpatients	Academic	RCT	40	√	√	≥ 18 years	10 weeks	Mobile-based APP
[42]	Brazil	2022	Analyzing the perceptions and satisfactoriness of a home-based exercise program in SLE and juvenile idiopathic arthritis (JIA) adolescent patients during COVID-19, and investigating the outcomes of the intervention on quality of life, sleep quality, and mental health conditions factors	Outpatients	Academic	RCT	51	√	–	10–19 years	12-week	Web-based exercise program
[37]	USA	2022	Assessing the feasibility and acceptability of a Web-based treatment program for lupus patients	Outpatients	Academic	Before-and-after study	83			18 years or older	TWO weeks	Web-based therapy program

**Fig. 2:**
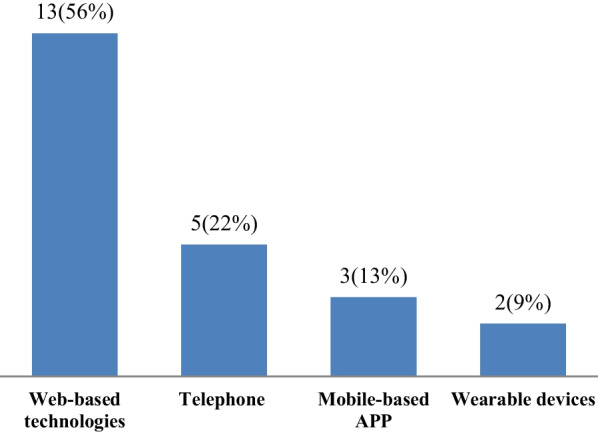
The most widely used health information technologies in the control, management, and monitoring of lupus

Figure 2 shows the most widely used health information technologies used in the control, management, and monitoring of lupus. About 75 percent of the studies used web-based technologies (n = 13) [22, 24, 25, 28, 29, 32, 33, 35, 37–39, 41, 42] and telephones (n = 5) [20, 26, 27, 30, 31] to control and manage lupus. The rest of the studies used mobile-based App (n = 3) [21, 34, 36] and wearable devices (n = 2) [23, 40] to control and manage the disease.

As it is shown in Tables 1 and 2, most of these technologies were conducted in the United States (n = 18) [20–37]. UK [38, 39], Canada [40], Thailand [41], and Brazil [42] were other countries that focused on technologies related to lupus control and management. The United States was the largest producer of web-based technologies (n = 9) [22, 24, 25, 28, 29, 32, 33, 37, 41], telephones (n = 5) [20, 26, 27, 30, 31], and mobile-based Apps (n = 3) [21, 34, 36]. The frequency and percentage of other technologies based on the geographical region are shown in Table 2.

**Table 2:** Types of lupus management and control technologies based on geographic region

Country	Type of information technologies			
	Web-based technologies (frequency)	Telephone (frequency)	Mobile-based APP (frequency)	Wearable devices (frequency)
USA	5	5	3	1
UK	2	0	0	0
Canada	0	0	0	1
Thailand	1	0	0	0
Brazil	1	0	0	0

Most of the services provided by these technologies were related to ‘training and consulting’ in various areas of lupus control and management. Then, ‘Self-reported physical and mental health status’ and ‘medication adherence reminders’ were the next services provided through information technologies for the control and management of lupus, respectively (Table 3).

**Table 3:** Types of services provided through information technologies

Services provided through information technologies	Refs.	Frequency
Training and consulting	[21, 26–28, 30–33, 35–38, 40–43]	16
Collection of demographic, clinical, and research data through electronic tools	[20, 21, 25, 34, 35, 39, 42]	7
Self-reported physical and mental health status	[22–24, 29, 32]	5
Medication adherence reminders (via SMS services, reminders, alerts, and email)	[21, 28, 34]	3
Measurement and recording of physical activity	[23, 40]	2
Symptom management and control	[21, 37]	2
Use a quiz/game to reduce anxiety and stress	[21]	1
Interaction between healthcare providers and patients	[35]	1

Table 4 shows the different outcomes of using health technologies in the management and control of lupus. As shown in this table, most technologies have led to ‘Better management and control of lupus. ‘Increasing knowledge and awareness of people about lupus’ and ‘Improving behaviors and attitudes of self-management and self-care’. Other outcomes are presented in Table 4.

**Table 4:** Different outcomes of using health Information technologies in the management and control of lupus

Outcomes	Refs.	Frequency
Better management and control of lupus	[21, 24, 29, 35–37, 40, 42]	8
Increasing knowledge and awareness of people about lupus	[21, 31, 33, 36, 40, 42]	6
Improving behaviors and attitudes of self-management and self-care	[25–28]	4
Increasing adherence to treatment	[28, 32, 34]	3
Increasing the quality of life	[36, 41, 42]	3
Improving the mental and physical health of people	[30, 39]	2
Increasing the level of physical activity	[23, 40]	2
Reducing the complications of the disease	[41]	1
Integration of information of a patient	[35]	
Increasing interaction between healthcare providers and patients	[35]	1
Identification of patients with lupus	[22]	1

Table 5 presents eight different axes related to lupus that are controlled and managed by health information technologies and the data elements of each axis. Among the eight axes, the two axes of ‘lifestyle’ (n = 11) and ‘consultation and education’ (n = 9) were the most widely used axes emphasized in the studies. The ‘Demographic’ (n = 3), and ‘risk factors and ‘complications’ (n = 3) were the least mentioned axes in the studies.

**Table 5:** Axes and data elements that can be controlled and managed by health information technologies

Axes	Data elements	Refs.	Frequency
Life style	Exercise, nutrition, sexual health, patient perceptions of care from the physicians, Daily Physical Activity, flexibility, and endurance, mobility, social support	[23, 24, 26, 29, 30, 35–37, 39, 40, 42]	11
Consultation and education	Stress relaxation techniques, coping (with pain and disease, other lupus symptoms, and interpersonal issues), depression, anxiety and stress, mentoring program, control over the illness, enhancing self-confidence, self-efficacy, self-monitoring, enhancing self-confidence, controlling embarrassement, shyness and unwelcomeness, family role interdependency, traveling outside one’s neighborhood, social support, emotional health, emotional stability and verbal communication skills, measuring psychological scales, loneliness, communication and discrimination skills inventories, action planning, relaxation techniques to cope with chronic pain, manage sudden increases in pain and other symptoms and reducing flares, interpersonal issues, mental health issues, cognition issues, employment, mood, self-management capacity, habitual behaviors, self-reported conditions, applied cognition-abilities, applied cognition general concerns, anger, education level, vitality, self-disclosure, social support habitual behavior, knowledge of health conditions and treatment, adopting a healthy lifestyle and exercise, nutrition, sexual health, physical activity, appropriate exercise, mobility, social support	[25, 26, 29, 30, 32, 36, 37, 41, 42]	9
Symptom	Skin discoloration and scaring, pain, fatigue, hair loss, pain intensity, sleep disturbance	[24–26, 29, 37, 40, 41, 44]	8
Cultural, social and economic issues	Perceived discrimination, perceived cultural competence of provider(s); income, perceived cultural factors, working or unemployed, socioeconomic characteristics, social role, ability to participate in social roles, career satisfaction, job control	[22, 24, 26, 29, 31, 32]	6
Medication	Medication-induced weight gain, misappropriate use of medications, antibiotics prescription, nonsteroidal anti-inflammatory drugs, cyclooxygenase2 (COX-2) inhibitors, oral and intravenous steroids, hydroxychloroquine, azathioprine, cyclosporine, oral and injectable methotrexate, mycophenolate mofetil (MMF), and oral and cyclophosphamide (CYC), current medications at baseline	[20, 24, 26, 30, 39]	5
Medical history	Medical history, type, and frequency of organ involvement, dialysis, and transplant, current medications at baseline, history of surgery, pregnancy, smoking, and alcohol use	[20, 24, 31, 32, 41]	5
Risk factors and complications	Concerns around hair loss, complications, fatalism-fear of complications and how that impacts the perception of survival, depression, sleep disturbance, time in sedentary behavior, smoking	[26, 30, 31]	3
Demographic	Age, gender, religion, education, income, and relationship status, race, ethnicity, age at lupus onset	[26, 31, 45]	3

Also, among all the axes, the ‘consultation and education’ axis had the highest number of data elements that can be used in health technologies. The ‘demographic’ axis also had the least data elements.

Table 6 shows the advantages of various information technologies. ‘Collecting patient data and information', 'Providing education and consultation services to patients', 'Patient-reported outcomes measurement', and 'Increasing patients' knowledge and awareness of their disease' were the most important advantages of various information technologies.

**Table 6:** Advantages of various technologies

Type of information technologies	Advantages of various technologies	Refs.	Frequency
Web-based technologies	Collecting patient data and information	[22, 29, 32, 33, 35, 38, 39, 41]	8
	Providing education and consultation services to patients	[25, 28, 33, 37, 38, 41, 42]	7
	Patient-reported outcomes measurement	[24, 29, 32, 39]	4
	Increasing patients' knowledge and awareness of their disease	[24, 33, 38, 42]	4
	Improving and empowering self-management by patients	[25, 28, 33]	3
	Increasing interaction between patients-patients and patients-clinicians	[28, 38, 42]	3
	Assisting the patient in making treatment decisions and diseases management	[25, 33]	2
	Providing quality information about lupus	[33, 38]	2
	Reducing racial/ethnic disparities in lupus-related health outcomes	[33]	1
	Estimating the incidence and prevalence of SLE more accurately	[22]	1
	Improving treatment process adherence	[28]	1
	Performing rehabilitation activities and exercises without the need to be in the office	[42]	1
Telephone	Collecting patient data and information	[20, 27, 30, 31]	3
	Self-reporting of data by patients	[20, 27, 30]	3
	Improving disease self-management	[26, 30]	2
	Receiving self-management educations	[26, 30]	2
Mobile-based APP	Improving disease self-management	[21]	1
	Increasing adherence to routine clinic visits and the treatment process	[34]	1
	Increasing interaction with clinicians	[21]	1
	Helping patients manage their medications and appointments independently	[21]	1
	Registration of patients' medical records and access to them	[21]	1
	Easy symptoms management	[21]	1
	Patient education	[36]	1
	Performing rehabilitation activities and exercises without the need to be in the office	[36]	1
	Reducing anxiety and stress	[21]	1
Wearable devices	Easy measurements of physical activity (PA)	[23]	1
	Self-reporting of physical activity data	[23]	1
	Estimating energy expenditure	[23]	1
	Improving physical activity participation and patient outcomes	[40]	1

Also, three disadvantages of various technologies were identified in the included studies. 'Slow internet speed' [24, 38] and 'Challenges and problems related to appearance and usability' [25] were two disadvantages of Web-based technologies. 'Patient concerns about privacy and misuse of their data' was another disadvantage of mobile-based APP.

Moreover, limitations for some technologies were mentioned in the included studies. 'Lack of information about research on new medications for SLE, yoga and meditation' [25], 'Need to access the Internet at a suitable speed' [24, 29], and the 'Need to improve the appearances and usability of the system' [25] were the main limitations of Web-based technologies. Impossibility to capture water activities was also the most important limitation of one of the wearable devices [23].

## Discussion

In this study, the role of health information technology in the control and management of Systemic Lupus Erythematosus was investigated. Web and telephone were the most widely used information technologies for controlling and management of SLE. ‘Training and consulting’ were also the most common services provided through health information technologies. Among the eight identified axes of' ''Life style', 'Consultation and education', 'Symptom', 'Cultural, social and economic issues', 'Medication', 'Medical history', 'Risk factors and complications' and 'Demographic', the two axes of ‘Lifestyle’ and ‘Consultation and education’ were the most widely used axes in the development of health information technologies. ‘Controlling and managing lupus’ was the most important outcome of using health information technologies for this disease.

As mentioned above, among the identified technologies, web-based technologies were the most widely used technologies in the control and management of SLE. Barak et al. [46] examined Internet-based interventions and concluded that due to the increasing acceptance of the Internet as a social communication tool and the continuous improvement of computer hardware and software (especially in terms of ease of use, privacy, and communication facilitation), the use of web services to control and manage diseases is increasing. Study of Toivonen et al. [47] have reported a rise in the use of IT for controlling and managing diseases due to reducing waiting times, improving quick and ubiquitous access to individual schedules (especially in asynchronous treatments), preserving confidentiality, and reducing healthcare costs [47].

Also, a systematic review and meta-analysis study conducted on caregivers showed that web-based technologies can significantly improve self-efficacy and self-esteem [48]. In the study by Wahbeh et al. [49], 71.2% of participants (365 out of 500) preferred to use web-based technologies for their treatment rather than in-person visits. Therefore, the advantages presented in the above studies, i.e. good acceptance of the Internet as a platform for interaction, continuous improvement and upgrade of computer hardware and software, easy and 24-h access [47], reduction of waiting time, reduction of costs, and improving self-efficacy and self-esteem [48] can be important reasons for the adoption of web-based technologies.

Other findings of our study showed that next to web-based technologies, telephone has been the most widely used technology in the control and management of lupus. Ristkari et al. [50] investigated the influence of a web-based education and telephone-coaching program of parents on disruptive behavior in 4-year-old children. The results showed this intervention increases feasibility, loyalty, accessibility and users’ satisfaction with medical services. Some studies have also shown that telephone interventions can increase patients' quality of life, improve their self-management behaviors and mental and physical functions [51], and increase their adherence to treatment. [52] Telephone support for self-management or disease management is a promising way to improve care for patients with chronic diseases. Inglis et al. [53] examined the effect of telephone intervention on heart disease and concluded that telephone intervention can reduce mortality and hospitalization of patients with heart failure. In addition, telephone use can enhance health-related quality of life, patients' knowledge and awareness, and self-care behaviors [53].

In this study, we found that ‘training and counseling’ in various areas of self-care and self-management were the services highly provided through health information technologies. Also, the data elements of the two axes of ‘Lifestyle’ (concerning exercise, nutrition, sexual health, patient perceptions of care from the doctor(s), daily physical activity, flexibility, and endurance, mobility, and social support) and ‘education and counseling’ were used more than elements of other axes, in the development of technologies. Dantas et al. [18] examined various applications for lupus control and management offered on Google Play and the App Store. They concluded that most applications focused on ‘training’ and then ‘symptom tracking’. Moses et al. [54] conducted a qualitative study on patients with lupus and concluded that patients mostly need education and counseling about their disease, proper lifestyle, continuity of health care, and sharing their experiences of the disease, respectively. However, despite providing several educational services and counseling related to lupus through different information technologies, the level of knowledge and awareness of lupus patients about this disease is low. Yang et al. [55], by examining the level of awareness of lupus patients about self-care processes and related factors, concluded that since patients with SLE have a moderate level of knowledge and awareness about different aspects of their disease, they need training in this regard [55]. As noted by Dantas et al. [18], despite development of many health information applications for the education, management, and control of lupus, existing technologies are currently of poor quality and have limited capabilities [18]. Therefore, before patients use a health information technology, its quality and capabilities should be ensured to increase its acceptance rate and continuously use. A quality technology with many capabilities can improve self-management and self-care attitudes of patients, resulting in better health.

'Sign' was the only axis that was not used in studies for the development of health information technologies. Lower use of Sign axis has several reasons. Thong et al. [56] stated that SLE is a challenging disease that is diagnosed, managed, and controlled with unique issues. The onset of the disease may be insidious and, despite many different signs and symptoms, early and accurate diagnosis is challenging for physicians [56]. On the other hand, the diagnosis of SLE is based on a combination of clinical manifestations, laboratory findings, serology, and histology of the affected organs (usually the skin and kidneys) and without these methods, the physician cannot diagnose it alone [57] and screening tests for SLE are not always useful [56]. Also, patients with lupus may need to see a dermatologist, nephrologist, neurologist, hematologist, or rheumatologist to control and manage their disease [56]. Fernando et al. [58] revealed that a combination of history taking, physical examinations, and laboratory tests such as hematology, biochemistry, urinalysis, and anti-dsDNA titers can be used to control lupus. Therefore, since lupus is an insidious disease and controlling its signs requires constant monitoring of specialized physicians of different orientations and performing different clinical tests, the Sign axis is the least used axis. Also, since most technologies focus on self-management and self-care processes by patients, they may not be as efficient in sign management because sign management need to be supported by different specialties.

Generally, the findings of most of the included studies have shown that health information technologies in the control and management of SLE can improve self-care and self-management. In this regard, some studies [59] have shown that, in addition to patient control and management, health information technologies can reduce human error, improve clinical outcomes, improve practice efficiencies and facilitate coordinated care and provide data tracking over time. Therefore, the use of digital technologies for control and management of various diseases is gradually increasing [60]. Health information technologies are developed to increase knowledge and individual abilities of people in the field of disease control and management. Improving knowledge and abilities can change people behavior, and eventually improve their health status [61].

According to other findings of the present study, ‘Collecting patient data and information’, 'Providing education and consultation services to patients', 'Measuring patient-reported outcomes, and 'Increasing patients' knowledge and awareness of their disease' were the most important advantages of various technologies. Also, 'Slow internet speed' and 'Challenges and problems related to appearance and usability', and 'Patient concerns about privacy and misuse of their data' were three disadvantages of technologies. Some studies have shown that information technology can be effective if it can collect and store patients' data and information [62], provide the possibility of training and counseling for patients [62, 63], and increase their knowledge and awareness about their disease [62–64]. Also, patients should be able to measure the outcomes related to their disease through information technology [62]. On the other hand, when the systems are faced with challenges such as slow or poor internet speed [65, 66], low usability [67, 68], and lack of privacy and security of patient data, the rate of patients using them decreases day by day [63, 64]. Therefore, to maximize the efficiency of the health information technologies and improve patient satisfaction, developers of these technologies are recommended to focus on the disadvantages and minimize the advantages, revealed by this study, during the development, implementation, updating, and maintain in these technologies.

### Novelty and limitations of the current work

Table 7 shows the novelty and limitations of the current work.

**Table 7:** Novelty and limitations of the current work

Limitations	Novelty
In this study, only studies in English were reviewed. If a study has been published in a language other than English, we may have missed it. Similar systematic reviews in future can also include articles published in other languages In the present study articles were searched in four scientific databases of Scopus, PubMed, Web of Science, and Embase. However, these databases are more likely to retrieve the most relevant articles. It is suggested that other studies to search a larger number of databases to achieve more comprehensive results Another limitation was that few studies did not report some of the required information. Although we contacted the authors of these studies via email, none of them responded to us Lack of critical assessment of the study quality and risk of bias assessment are other limitations of this review that future studies can focus on	This is the first study that systematically reviewed the use of information technology to control and manage lupus disease. The results can be used as a basis for other studies This study identified the most widely used health information technologies for controlling, management, and monitoring lupus, types of lupus management and control technologies based on geographic region, and types of services provided through technologies Identifying different outcomes of using health information technologies for the management and control of lupus, and specifying axes and data elements that can be controlled and managed by health information technologies are other strengths of this study

## Conclusion

Due to the lack of evidence about the use of health information technology for controlling and managing lupus, in this systematic review, the role of these technologies in the control and management of lupus was investigated in four databases. Among different information technologies, web and telephone-based technologies were the technologies widely used for controlling and management of lupus, respectively. ‘Training and consulting’, ‘Collecting demographic, clinical and research data through electronic tools’, and ‘Self-reported physical and mental health statuses were the most common services provided by these technologies, respectively. The most important outcome of using these technologies was ‘Better management and control of lupus’. Among the eight axes identified in this study, two axes of ‘lifestyle’ and ‘Consultation and education’ were known as the most widely used axes in the development of health information technologies.

Patients with lupus can easily control and manage their disease using the capabilities provided by health information technology. Beside accessing health information, these information technologies increase people's knowledge and awareness about lupus, educate patients and doctors, improve self-management and self-care behaviors and attitudes, improve mental and physical health of people, increase patients' adherence to treatment, reduce the complications of the disease, and increase the quality of life. The findings of our study can inform the development of future interventions and their adoption for controlling and management of lupus. The outcomes of this study can be the basis for developing and implementing efficient information systems to improve, control and manage people with lupus. Moreover, based on the findings of this study, health information technology designers can develop high-quality and safe technology that may result in saving their time and cost.

## Data Availability

All data generated or analysed during this study are included in this published article.

## Abbreviations

SLE: Systemic lupus erythematosus
HIT: Health information technology
RCT: Randomized controlled trial
eGFR: Estimated Glomerular Filtration Rate
COX-2: Cyclooxygenase2
MMF: Mycophenolate mofetil
CYC: Oral and cyclophosphamide
